# Human Rabies Cluster Following Badger Bites, People’s Republic of China

**DOI:** 10.3201/eid1312.070465

**Published:** 2007-12

**Authors:** Gong Zhenyu, Wang Zhen, Chen Enfu, He Fan, Lin Junfen, Li Yixin, Ding Gangqiang, R.E. Fontaine

**Affiliations:** * Zhejiang Provincial Center for Disease Control and Prevention, Zhejiang, People’s Republic of China; † Chinese Center for Disease Control and Prevention, Beijing, People’s Republic of China; ‡ Chunan Center for Disease Control and Prevention, Chunan, Zhejiang, People’s Republic of China

**Keywords:** rabies, badger, prevention, control, human, letter

**To the Editor:** From February 2002 to April 2004, 7 rural residents of Coteau County (population 450,000) in western Zhejiang Province in eastern People’s Republic of China died of rabies following badger bites (Figure). In this county, 89% of residents are farmers. The county covers 4,475 km^2^, and the terrain is mountainous. No other cases of human rabies had been reported from this county since 1986. We investigated the cluster to ascertain characteristics of these exposures.

**Figure:**
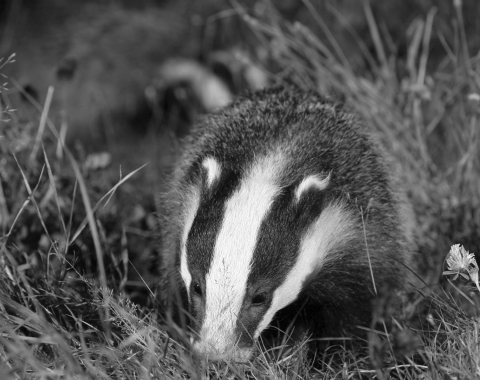
Badger: a new natural reservoir of human rabies? (Image source: Ian Stickland)

Rabies testing was not readily available. In China, the national case definition is based on clinical compatibility with appropriate animal exposure. Doctors are required to report rabies according to a general case description published by the Ministry of Health. Laboratory confirmation is not generally performed. We defined a rabies case as any person from Coteau County in whom rabies was diagnosed by a physician from February 2002 through March 31, 2007. We interviewed family members of case-patients and neighbors about the characteristics of the illness and activities associated with badgers, dogs, and other animals that are potential rabies reservoirs.

From February 2002 to April 2007, a total of 8 human rabies cases were reported from Coteau County. Seven case-patients had badger exposure and 1 had cat exposure. Badger-associated rabies occurred from February 2002 to December 2004; 1- to 2-month intervals generally occurred between cases. The average yearly incidence rate for human rabies in the county was 0.52 per 100,000 compared to 0.15 per 100,000 for China for the same period. Patients ranged in age from 18 to 76 years (mean 54 years). Badger-associated rabies was confined to 7 contiguous townships in the center of the county. Signs and symptoms were typical of rabies, namely, fever, excitation, aerophobia, hydrophobia, dysphagia, and hypersalivation, leading to coma and death. Incubation periods ranged from 31 to 100 days (mean 45 days).

All 7 case-patients with badger-associated rabies had tried to catch badgers that were sluggish and could not escape. All bites occurred on the fingers, when the badger was captured or carried home. The captors killed and ate 2 badgers, 4 badgers died spontaneously, and the fate of 1 badger was not known. The cat-associated rabies case from the same area occurred in February 2004. The cat died spontaneously during the same period when some badgers died spontaneously nearby. We found no other villagers who had been bitten by these or other badgers. The case-patients and family members did not know that badgers can transmit rabies and did not seek treatment or postexposure prophylaxis. These case-patients had no other exposure to bites from other potentially rabid animals in the 10 years before onset.

The 7 case-patients lived in villages covering an area of ≈10 km^2^, representing ≈0.2% of the total county area. The individual villages were 1,500–3,000 m apart. All were on the same side of a mountain ridge. Mountainous terrain and limited transportation isolate this county from nearby counties. Villagers reported seeing dead badgers before human cases occurred. During the past 20 years in this county, ≈15,000 persons received rabies postexposure prophylaxis after dog bites, but no rabies occurred. During 2002–2004, no human rabies cases followed exposure to dogs that were within 50 km of this county.

After 2004, we set up a rabies surveillance and health education system in this county. At the end of 2004, we advised the public in this and 5 neighboring counties to avoid catching and killing badgers and, if bitten, to seek postexposure prophylaxis. Since that recommendation, no human rabies has occurred in the area. In 2006, a total of 1,719 residents were treated for animal bites. The incidence of animal exposures in this county is higher than in the United States (*1*). Dog bites accounted for 86% (1,471), cat bites for 9.5% (164), and other animals for 4.9% (84) of exposures. However, no badger bites were reported.

We concluded that an epizootic of badger rabies affected a limited area of Coteau County from 2002 through 2004. Badgers can easily transmit the virus and could be an important secondary host of rabies (*2*). Research is needed on badgers as a natural reservoir of human rabies and on control of this disease in wildlife hosts (*3*,*4*). A national surveillance system for animal rabies should be set up in this region (*5*).

A major limitation of this study is the lack of laboratory support for surveillance of both human and animal rabies. Accordingly, we based our conclusion on clinical and epidemiologic histories. The lack of human cases from dogs could be attributed to effective postexposure prophylaxis of humans following dog bites. On the other hand, rabies following dog bites is the number-one cause of death from infectious diseases in China, in part because of absent or incomplete postexposure prophylaxis for poor rural residents. Thus, the complete absence of reported dog-associated rabies is unusual. China is planning increased investment in rabies surveillance and prevention that will include recommended laboratory support and should help alleviate this situation in the future (*3*).
